# Serotype distribution and antimicrobial susceptibility of *Streptococcus pneumoniae* isolates from a Phase III community-acquired bacterial pneumonia (CABP) trial

**DOI:** 10.1093/jacamr/dlab057

**Published:** 2021-05-04

**Authors:** S P McCurdy, A J Sheets, S K Cammarata, J E Vidal

**Affiliations:** Melinta Therapeutics, Morristown, NJ, USA; Melinta Therapeutics, Morristown, NJ, USA; Melinta Therapeutics, Morristown, NJ, USA; Emory University, Atlanta, GA, USA

## Abstract

**Objectives:**

To report *Streptococcus pneumoniae* serotyping and susceptibility data from a recent clinical trial (ML-3341-306) comparing delafloxacin with moxifloxacin in the treatment of adults with community-acquired bacterial pneumonia (CABP).

**Methods:**

Serotyping and susceptibility testing were conducted on 142 baseline *S. pneumoniae* isolates recovered from subjects participating in a CABP clinical trial.

**Results:**

Overall, 113/142 (79.6%) isolates were vaccine serotypes. 76.8% (109/142) of serotyped isolates were PPSV23 serotypes and 59.9% (85/142) of isolates were PCV13 serotypes. 15.5% (22/142) of serotyped isolates were serotypes not covered by either vaccine; 4.9% (7/142) of tested isolates were non-typeable. The most common serotypes were serotypes 3 (19.0%; 27/142), 19F (9.9%; 14/142) and 23F (7.0%; 10/142). All of the 142 isolates were susceptible to delafloxacin and moxifloxacin, 76.1% were susceptible to azithromycin and 71.8% were susceptible to penicillin. Multidrug resistance was found among 19A (4/5; 80%), 6A (1/4; 25%), 6B (1/4; 25%), 14 (1/4; 25%), 19F (1/14; 7.1%), and 23F serotypes (2/10; 20%), and among non-typeable *S. pneumoniae* isolates (1/7; 14.3%).

**Conclusions:**

*S. pneumoniae* vaccine-targeted serotypes were the main cause of CABP in this Phase 3 CABP study. Fluoroquinolones including delafloxacin remain a good treatment option for CABP in adults caused by *S. pneumoniae*.

## Introduction 

Community-acquired bacterial pneumonia (CABP) is a major cause of morbidity and mortality worldwide, especially in older age groups.^1^ The predominant pathogen causing CABP is *Streptococcus pneumoniae,* but this pathogen can also invade normally sterile body sites such as the blood and meninges resulting in invasive pneumococcal disease (IPD).^2^ There are two types of polysaccharide vaccines available for preventing pneumococcal infections in adults. The 13-valent pneumococcal conjugate vaccine (PCV13) comprises 13 serotypes (1, 3, 4, 5, 6A, 6B, 7F, 9V, 14, 18C, 19A, 19F and 23F) and has been licensed in Europe since 2011 and in the US since 2012 for adults aged ≥50 years.^3^^,^^4^ The 23-valent pneumococcal polysaccharide vaccine (PPSV23) comprises 23 serotypes and covers 11 serotypes not found in PCV13 (2, 8, 9N, 10A, 11A, 12F, 15B, 17F, 20, 22F and 33F).^5^ This vaccine has been available since 1983 but is reported to be less immunogenic, especially in older adults.^6^

In a 2019 United States (US) CDC report, US coverage of adults ≥65 years with any pneumococcal vaccine type was 62% (45% received PPSV23 and 30% received both PCV13 and PPSV23).^7^ In Europe, the use of PCV13 [with or without additional dose(s) of PPSV23] is favored.^3^ While the introduction of conjugated pneumococcal vaccines in both children and adults resulted in a significant decrease in IPD in adults ≥65 years in the US, this reduction in incidence stalled between 2014–17 and no further reduction in PCV13-type IPD incidence was observed among adults age ≥19 years in the US.^7^

Further, antibiotic resistance among *S. pneumoniae* isolates has been increasing. The commonly used macrolide class of antibiotics has been associated with high levels of pneumococcal resistance, approaching 50% in areas of North America and 66.8% in Asia.^8^^,^^9^

Delafloxacin is a novel fluoroquinolone antibiotic that possesses Gram-positive, Gram-negative, and atypical activity including activity against fluoroquinolone non-susceptible MRSA isolates.^10^ It offers flexibility of intravenous and oral treatment with no QT restrictions or phototoxicity^11^ as well as no major drug–drug interactions.^12^ Delafloxacin is FDA and EMA approved for treatment of patients with acute bacterial skin and soft tissue infections (ABSSSI) and CABP.^12^ The microbiological results of the Phase 3 CABP trial were previously reported,^13^^,^^14^ high eradication rates were observed for delafloxacin (92.7%) and moxifloxacin (93.9%) for patients with *S. pneumoniae*. In a Phase 3 CABP trial completed in 2018 comparing delafloxacin with moxifloxacin, *in vitro* susceptibility results for delafloxacin and comparator agents as well as *S. pneumoniae* serotypes were determined and are reported here.

## Materials and methods

### Microbiology

*S. pneumoniae* was isolated by culture of baseline specimens including sputum, bronchoalveolar lavage (BAL), nasopharyngeal (NP) swabs, or blood. For *S. pneumoniae* cultured from NP swabs, a concomitant *lytA* PCR value of ≥1000 gene copies/mL was required for the isolate to be considered a pathogen.^15^^,^^16^ Additional information regarding study design, efficacy endpoints, analysis sets, microbiological outcomes as well as a comparison of the diagnostic method yield for *S. pneumoniae* was previously reported.^13^ All isolates underwent susceptibility testing and serotyping (see below).

### Susceptibility testing

Isolates were submitted to the central laboratory (Covance Laboratories, Indianapolis, IN, USA) for identification confirmation and susceptibility testing according to CLSI guidelines.^17^ For suspected *S. pneumoniae* isolates, optochin disc testing was performed. If the result was ≥10 mm, a bile solubility test was performed. If the result was <10 mm and/or negative for bile solubility, MALDI-TOF identification was employed. If the MALDI-TOF identification was found to be inadequate, a secondary identification system such as a Vitek 2 streptococci panel, API^®^ Rapid ID 32 STREP strip (bioMérieux, Marcy l’Etoile, France), and/or manual biochemical(s) were employed. For delafloxacin, susceptibility interpretative criteria were applied using FDA breakpoints.^18^ For all comparators, CLSI susceptibility interpretative criteria were applied.^19^ Multidrug resistance was determined using the CLSI susceptibility interpretative criteria and as previously described.^20^

### lytA PCR assay

The *lytA* PCR assay is a laboratory-developed test that targets the autolysin gene *lytA*, a single-copy gene that is carried by all pneumococcal strains.^15^^,^^16^ Sequences of the primers and probe and assay conditions were previously described, with NP swabs used as specimen types.^21^

### S. pneumoniae serotyping

All *S. pneumoniae* isolates were serotyped by the Quellung reaction using Neufeld reagents (Statens Serum Institute, Copenhagen, Denmark) at Emory University. Non-typeable isolates were also tested by latex agglutination and confirmed to be non-typeable using Quellung antisera (results not shown). If the same serotype was isolated from multiple specimen types from the same subject, it was counted only once. If multiple serotypes were isolated from multiple specimen types from the same subject, an isolate serotype recovered from a BAL, sputum or blood specimen type was counted over an isolate serotype recovered from a nasopharyngeal swab specimen type.

## Results

### Patient demographics with culture-positive S. pneumoniae

A total of 859 adult patients from sites on four continents, including sites in the United States (0.7%), Europe (85.7%), Latin America (5.4%), and South Africa (8.3%), were enrolled. Patients had to meet entry criteria and to have radiological evidence as well as ≥2 clinical signs and symptoms of CABP, including cough, production of purulent sputum consistent with a bacterial infection, difficulty breathing (dyspnoea), and chest pain due to pneumonia. In addition, multiple laboratory diagnostic methods were employed including respiratory, or blood culture, PCR, serology, and urinary antigen tests. Using this methodology, (181/520; 34.8%) subjects in the microbiological ITT population had a definite diagnosis of CABP caused by *S. pneumoniae.*^13^^,^^22^

Among subjects with culture-positive *S. pneumoniae*, 65% (92/142) were male. In comparison, in the CABP study population overall, the percentage of subjects that were male was 57%.^22^ Among study participants with pneumococcal disease, the mean age was 57 years (range: 18–86 years). 37% of the subjects were ≥65 years of age (study population overall: 44.5%)^22^. 81.7% (*n = *116) of the subjects with culture-positive *S. pneumoniae* were from Eastern Europe, which is similar to the overall geographic enrolment in the trial; the three most-represented countries overall were Ukraine (*n = *24), Serbia (*n = *23), and Romania (*n = *19). It has been reported that Eastern European clinical trial sites enrol more subjects per site than North American sites, which was consistent with the enrolment observed in this clinical trial.^23^

### Serotyping of S. pneumoniae strains

Of the 142 unique *S. pneumoniae* isolates, 135 (95.1%) were typeable and 38 different serotypes were identified. Thirty-five paired isolates were recovered from the NP swab and another source (sputum, blood or bronchoalveolar lavage), with 33 cases (94.3%) having an identical serotype. All 35 (100%) patients that had paired isolates also had a *lytA* qPCR result that met the pre-specified diagnostic threshold of ≥1000 genome copies/mL.^15^^,^^16^

**Figure 1.**
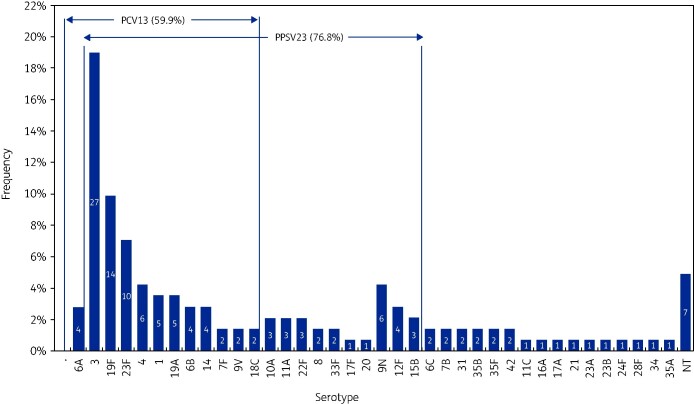
Serotype distribution of baseline *S. pneumoniae* isolates.

Overall, 120/142 (84.5%) isolates were vaccine serotypes. 76.8% (109/142) of serotyped isolates were PPSV23 serotypes and 59.9% (85/142) of isolates were PCV13 serotypes. 15.5% (22/142) of serotyped isolates were serotypes not covered by either vaccine; 4.9% (7/142) of tested isolates were non-typeable. The most common serotypes were serotypes 3 (19.0%; 27/142), 19F (9.9%; 14/142) and 23F (7.0%; 10/142) (Figure 1).

Overall, 66.2% (94/142) of the serotyped *S. pneumoniae* isolates were recovered from monomicrobial infections. Notably, of the three most common serotypes, a high proportion of 19F serotypes were isolated from a monomicrobial infection (92.9%; 13/14) while serotype 3 (63.0%; 17/27) and serotype 23F (40%; 4/10) were frequently isolated from polymicrobial infections. One serotype 3 and one serotype 23F were recovered from blood or bronchoalveolar lavage while no 19F serotypes were recovered from these sample types (Table 1).

**Table 1.** Delafloxacin and comparator activity against *S. pneumoniae* serotypes^a^ from blood and bronchoalveolar lavage

Antibiotic	MIC_50_ (mg/L)	MIC_90_ (mg/L)	MIC range (mg/L)	Susceptible (%)
Delafloxacin	0.015	0.015	0.008–0.015	100
Moxifloxacin	0.25	0.25	0.12–0.25	100
Azithromycin	0.25	0.25	0.12–>32	90.9
Penicillin^b^	≤0.03	≤0.03	≤0.03	100
Clindamycin	0.06	0.12	0.06–>32	90.9
Ceftriaxone	0.015	0.03	0.015–0.03	100

a The *S. pneumoniae* serotypes and numbers of isolates were: 9N (*n = *1); 18C (*n = *1); 4 (*n = *3); 1 (*n = *2); 9V (*n = *1); 23F (*n = *1); 3 (*n = *1); and 12F (*n = *1).

b CLSI oral penicillin breakpoints.^20^

### Susceptibility testing results

For all 142 *S. pneumoniae* isolates, 100% were susceptible to delafloxacin and moxifloxacin, 76.1% were susceptible to azithromycin and 71.8% were susceptible to penicillin. For serotype 3, all isolates were susceptible to delafloxacin, moxifloxacin, azithromycin, and penicillin. In contrast, for serotype 19F, 85.7% of isolates were susceptible to azithromycin and only 14.3% were susceptible to penicillin. For serotype 23F, 40% (4/10) were susceptible to azithromycin and 50% (5/10) were susceptible to penicillin (Table 2). Multidrug resistance was found among 19A (4/5; 80%), 6A (1/4; 25%), 6B (1/4; 25%), 14 (1/4; 25%), 19F (1/14; 7.1%), 23F serotypes (2/10; 20%) and among non-typeable *S. pneumoniae* isolates (1/7; 14.3%). For isolates that were recovered from BAL or blood, greater susceptibility was observed compared with the *S. pneumoniae* isolates overall, however, this finding may be the result of the small number of isolates recovered from these sample types (Table 1).

**Table 2.** Delafloxacin and comparator activity against *S. pneumoniae* serotypes

Serotype/antibiotic	MIC_50_ (mg/L)	MIC_90_ (mg/L)	MIC range (mg/L)	Susceptible (%)
All *S. pneumoniae* serotypes (*n = *142)				
Delafloxacin	0.015	0.015	0.004–0.03	100
Moxifloxacin	0.25	0.25	0.06–0.25	100
Azithromycin	0.25	>32	0.06–>32	76.1
Penicillin^a^	≤0.03	2	≤0.03–4	71.8
Clindamycin	0.06	>32	≤0.015–>32	85.2
Ceftriaxone	0.015	0.5	≤0.008	100
*S. pneumoniae* serotype 3 (*n = *27)				
Delafloxacin	0.015	0.03	0.008–0.03	100
Moxifloxacin	0.25	0.25	0.12–0.25	100
Azithromycin	0.12	0.25	0.06–0.25	100
Penicillin^a^	≤0.03	≤0.03	≤0.03–0.06	100
Clindamycin	0.06	0.06	≤0.015–0.12	100
Ceftriaxone	0.015	0.015	≤0.008–0.015	100
*S. pneumoniae* serotype 19F (*n = *14)				
Delafloxacin	0.015	0.015	0.004–0.03	100
Moxifloxacin	0.12	0.25	0.12–0.25	100
Azithromycin	0.25	>32	0.12–>32	85.7
Penicillin^a^	0.25	2	≤0.03–2	14.3
Clindamycin	0.06	0.5	0.06–>32	85.7
Ceftriaxone	0.06	0.5	0.015–1	100
*S. pneumoniae* serotype 23F (*n = *10)				
Delafloxacin	0.015	0.015	0.004–0.015	100
Moxifloxacin	0.12	0.25	0.06–0.25	100
Azithromycin	2	>32	0.25–>32	40
Penicillin^a^	≤0.03	2	≤0.03–2	50
Clindamycin	0.06	>32	0.06–>32	80
Ceftriaxone	0.12	1	0.015–1	100

a CLSI oral penicillin breakpoints.^20^

## Discussion

In this Phase 3 clinical trial, *S. pneumoniae* strains were identified as the aetiology of CABP in 43.5% of adults in the microbiological ITT population.^22^ Notably, in the ITT population only 1.2% (10/859) of subjects were vaccinated with a pneumococcal vaccine within 5 years of enrolment, although PCV13 versus PCV23 vaccination data were not collected. Consistent with this low vaccination rate, most of the recovered pneumococcal strains (84.5%) belonged to vaccine serotypes. These findings reflect variation in pneumococcal vaccine policies and availability across countries as well as where clinical trial sites were located. Adults carrying vaccine types may represent a source for the transmission of *S. pneumoniae* to children and/or to other susceptible adults. Certainly, vaccination with pneumococcal vaccines may help reduce the burden of pneumococcal CABP by vaccine-targeted serotypes in such countries.

It is notable that 100% of the *S. pneumoniae* isolates were susceptible to both delafloxacin and moxifloxacin and no fluoroquinolone-resistant isolates were recovered. In a recent surveillance study of CABP pathogens, >98% of *S. pneumoniae* isolates from Eastern and Western Europe, Asia and Latin America were susceptible to levofloxacin.^24^ In contrast to the situation with fluoroquinolones, reduced susceptibility to macrolides and penicillin were observed. β-Lactam and macrolide antibiotics are frequently utilized to treat pneumococcal disease in the USA and Europe.^25^^,^^26^ In our study, resistance to these first line antibiotics was particularly evident for serotypes 19F and 23F, with a proportion of these serotypes being classified as MDR.^20^ These two serotypes have previously been reported to be MDR from surveillance studies conducted in Canada,^27^ China^28^ and Qatar.^29^ Most macrolide resistance in *S. pneumoniae* is mediated by the efflux/ribosomal protection gene cassette, *mef*(E)/*mel* or the target-modifying RNA methylase gene, *erm*(B).^30^^,^^31^ The *mef*(E)/*mel* machinery confers resistance to 14- and 15-membered macrolides (M-phenotype); while *erm*(B) confers resistance to macrolides, lincosamides, and streptogramin B (MLS_B_ phenotype).^32^^,^^33^ These genes are often carried on transposons and thus, increasing resistance to macrolides in *S. pneumoniae* can be expected over time.

In conclusion, *S. pneumoniae* vaccine-targeted serotypes were the main cause of CABP in this Phase 3 CABP study. Fluoroquinolones including delafloxacin remain a good treatment option for CABP in adults caused by *S. pneumoniae*. However, one limitation of this study was the limited geographic distribution of *S. pneumoniae* isolates recovered from the clinical trial.
